# A Code Division Design Strategy for Multiplexing Fiber Bragg Grating Sensing Networks

**DOI:** 10.3390/s17112508

**Published:** 2017-11-01

**Authors:** Andrés Triana, Daniel Pastor, Margarita Varón

**Affiliations:** 1 Photonics Research Labs (PRL), Universitat Politècnica de València, 46022 Valencia, Spain; catrianai@unal.edu.co; 2 Electric and Electronics Department, Universidad Nacional de Colombia sede Bogotá, Bogotá D.C. 111321, Colombia; gmvarond@unal.edu.co

**Keywords:** fiber Bragg grating, FBG, encoding, overlap proof, optical fiber sensor, sensing network

## Abstract

In this paper, an encoding strategy is used to design specialized fiber Bragg grating (FBG) sensors. The encoding of each sensor requires two binary codewords to define the amplitude and phase patterns of each sensor. The combined pattern (amplitude and phase) makes each sensor unique and therefore two or more sensors can be identified under spectral overlapping. In this way, we add another dimension to the multiplexing of FBG sensors, obtaining an increase factor ‘*n*’ to enhance the number of sensors that the system can handle. A proof-of-concept scenario with three sensors was performed, including the manufacturing of the encoded sensors. Furthermore, an interrogation setup to detect the sensors central wavelength was proposed and its working principle was theoretically developed. Results show that total identification of the central wavelength is performed under spectral overlapping between the manufactured sensors, achieving a three-time improvement of the system capacity. Finally, the error due to overlapping between the sensors was assessed obtaining approximately 3 pm, which makes the approach suitable for use in real measurement systems.

## 1. Introduction

Optical fiber sensing technologies take advantage of optical fiber properties such as immunity to interferences, low losses, and light weight, among others [1]. Therefore, there has been great interest and development in optical fiber sensors and their multiplexing over the last decades. One of the most mature approaches in the field is the use of fiber Bragg grating (FBG) devices as sensors [2]. FBG devices are basically narrowband filters constructed from a periodical perturbation of the optical fiber refractive index. The period of the perturbation determines the reflected wavelength peak according to the equation $\lambda_{B}=2\eta_{eff}\Lambda$, where $\lambda_{B}$ is the Bragg wavelength, $\eta_{eff}$ is the effective refractive index of the fiber, and Λ is the grating perturbation period. Hence, the usual multiplexing approach for FBG sensors consists of the wavelength division multiplexing (WDM) technique [3]. By simply writing each FBG sensor at a different central wavelength, each sensor and its operational range can be assigned to a finite spectrum region, preventing the overlapping between adjacent sensors. Another common multiplexing approach is time domain multiplexing (TDM), which acquires signal spread in time, but usually requires a fast system with a pulsed signal and synchronization to distinguish each sensor contribution in the time domain. Although the most accepted FBG sensors are single FBGs, Bragg devices with multiple reflection bands can be custom-manufactured according to almost any required design. Superimposed FBGs have been demonstrated in silica fiber, mainly for code division multiple access (CDMA) applications and dense WDM multiplexing [4,5,6,7]. Another approach consists of the manufacture of super-structured fiber Bragg grating (SSFBG) devices, which have a complex index profile manufactured point by point until the custom spectrum is obtained [8,9,10], instead of a summation of single FBGs in the same location of the optical fiber.

Given that FBG sensors are naturally written at a specific wavelength, their multiplexing schemes rely ultimately on the wavelength range assigned to each sensor in the network. Therefore, the maximum number of sensors allocated in an array is limited by the total spectrum available and the working range defined for each sensor. Hence, the purpose of this study is to determine an encoding scheme that enables multiple FBG devices to work in the same spectral range. In other words, an encoding-based multiplexing approach is proposed to allow overlapping between neighboring sensors, so the number of sensors distributed in a single array can be improved a number of times ‘*n*’ by using ‘*n*’ codewords. We take advantage of the technological readiness to manufacture super-structured FBG devices with complex index profiles in phase and amplitude. In fact, we have previously demonstrated the possibility of manufacturing amplitude-encoded FBG sensors with orthogonal properties between them to achieve an enhancement in the number of sensors of a conventional interrogating scheme. Here, we propose the manufacture of specialized FBG sensors encoded using their amplitude and phase terms. The design of the proposed sensors is customized to fit the dual wavelength interrogating setup presented in [11], i.e., the device sub-bands are spaced according to the dual wavelength separation in the source. In this way, the relative phase between two adjacent sub-bands in the sensing network can be measured. Other interrogation approaches based on a dual wavelength source have been proposed in the past [12]. However, the difference with our approach is that we use the dual wavelength source to extract not only the amplitude but also the network phase pattern. We focus in the development of an additional multiplexing dimension (code multiplexing) to enhance the number of sensors in the network. Other approaches have been suggested to identify the central wavelength of FBG sensors under overlapping conditions [13,14,15,16], but these methods are computationally consuming and non-deterministic since they rely on bio-inspired computational techniques. The rest of the paper is organized as follows: in Section 2 the fundamental concepts regarding the sensor design and their interrogation method are considered. Section 3 addresses practical aspects of the measurement system such as the wavelength detection process based on the correlation between the expected and measured signals, and discusses the practical considerations taken into account to manufacture the proposed sensors. Later, in Section 3.3, the experimental validation of a measurement system with three encoded sensors sharing the spectrum is demonstrated. Finally, some conclusions are drawn in Section 4.

## 2. Principle

This section details the design process followed to generate a set of super-structured FBG (SSFBG) sensors encoded in amplitude and phase. In this way, a technical formulation of the proposed devices is provided, as well as the considerations taken into account to achieve the wavelength interrogation of each sensing element in the network.

There are two important factors in the proposed sensing system; the first one is the design of orthogonal codewords, composed of amplitude and phase terms, that allows a set of neighboring sensors to overlap in the spectrum, and the second one is the proper interrogation of the SSFBG devices to obtain the complete information from the sensing network. These two factors are closely related so it is necessary to conceive the whole sensing system to propose a feasible encoding scheme for the proposed SSFBG sensors.

### 2.1. Design

The super-structured FBG devices are designed as a set of reflection sub-bands equi-spaced in wavelength. The sub-band sets that comprise an SSFBG are written at once in the same location along the optical fiber. Therefore, the entire SSFBG device responds to environmental changes uniformly, i.e., all the sub-bands are wavelength-shifted to the same extent $\Delta \lambda_{B}$. Each one of the sub-bands comprising the SSFBG device is manufactured according to the amplitude and phase codewords.

Encoding sensors in amplitude is somewhat straightforward: a binary codeword can be translated into the design of a super-structured FBG sensor by activating/deactivating each reflection sub-band depending on a corresponding bit value (‘1’ or ‘0’). Take for example a codeword composed of five bits [0 1 0 0 1]. This codeword would be translated to a SSFBG device composed of five equidistant wavelength slots featuring reflection sub-bands only in the second and fifth positions (corresponding with the 1’s in the codeword). Phase information can also be assigned to each active sub-band, i.e., each reflection sub-band is able to phase-shift the reflected waves by a fixed value, with the property that the relative phase between two consecutive sub-bands is fixed to a discrete value (e.g., ‘0’, ‘π’). Given that the phase relationship is preserved between consecutive sub-bands in each sensing device, the interrogation method should measure the differential optical phase along the sensing network.

A feasible way to measure the phase information encoded into the sensors in a scanning fashion (common to interrogation methods) is through comparison of the phase value between two adjacent sub-bands, obtaining the relative phase-shift through comparison to a local oscillator. This is the reason why single-sideband (SSB) modulation of the source is performed, obtaining a tunable dual-wavelength source interrogation system. The key of this interrogation scheme is that the dual-wavelength source separation matches the distance between sub-bands in the sensing device, so that the relative phase between two adjacent sub-bands in the sensing network can be measured. This makes it possible to distinguish whether or not two adjacent sub-bands introduce a phase shift.

**Figure 1:**
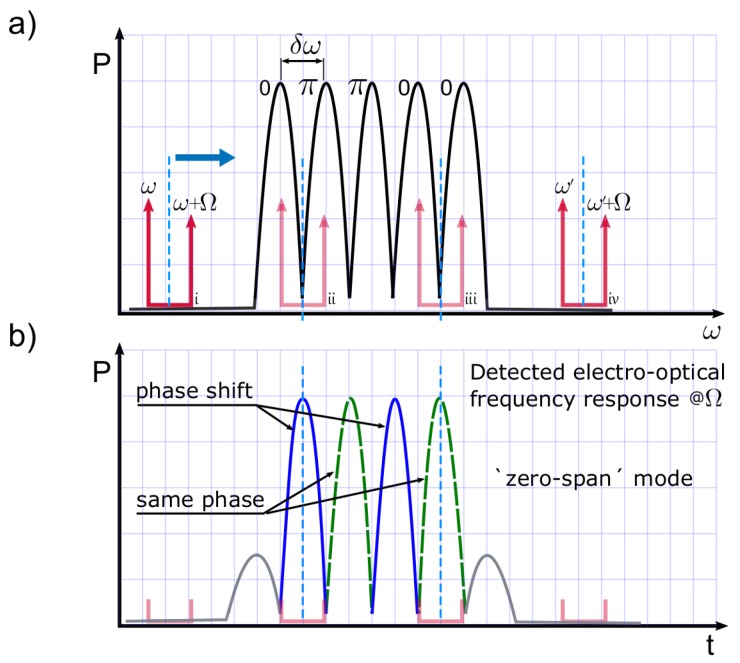
Illustration of the interrogation method used to retrieve the frequency response of the sensing network in phase and amplitude. In (**a**), the dual-wavelength source scanning process is depicted; (**b**) depicts the electro-optical frequency response for the sensing device; amplitude and phase response from the sensing array are obtained.

Figure 1 exemplifies a SSFBG device and shows the operating principle for its interrogation. Figure 1a, represents the scanning of the dual-wavelength source over a super-structured FBG device with five active reflection sub-bands a=[11111]. Let us consider that their optical phase values correspond with the following codeword: f=[0ππ00]. The spectral spacing between sub-bands (δω) is fixed to match the spectral distance Ω between the two tones of the tunable dual-wavelength source used to interrogate the sensors. In addition, Figure 1b shows the expected amplitude and phase values of the radiofrequency-detected signal at Ω, after photo-detection of the interaction between the dual-wavelength source and the sensing device (as exemplified in Figure 1a). The scanning interrogation method produces a *interrogated sub-band* for each consecutive pair of sub-bands in the actual SSFBG device, with a relative phase value depending on the phase relationship between sub-bands in the original device. The interrogation process is depicted in Figure 1a in four stages: stages i and iv represent the starting and ending points of the scan, respectively, while stages ii and iii represent two detection possibilities: differential phase shift of π, or no phase change at all, respectively.

From the establishment of super-structured FBG sensors and their interrogation methodology discussed above, we move now to describe the design of mutually orthogonal super-structured FBG sensors.

The first consideration to get into the design of the SSFBG sensors has to do with the practical parameters needed to manufacture the devices. Each SSFBG sensor shape in the network is established by a number of parameters that can be optimized either to facilitate their detection or to adapt the sensors behavior to the network characteristics (e.g., network topology could require higher/lower reflectivity values). The main descriptor parameters in our SSFBG sensors are of course the amplitude and phase codewords that define sub-band distribution. Still, other important design parameters are the spacing between slots δλ (which is related with the frequency spacing of 10 GHz by $\delta \nu =\frac{c}{\lambda^{2}}\delta \lambda$) and the sub-band linewidth $\Delta \lambda_{{}_{FWHM}}$, as described in Equation (1) for a set of *K* encoded SSFBG sensors.

(1) $$\begin{array}{cc} R\left(\lambda \right)=\sum_{k=1}^{K}\;\; & \sum_{p=-N/2+1}^{N/2}a_{k}\left(p\right)\cdot \bar{R}\cdot \exp\left(j\pi \cdot f_{k}\left(p\right)\right)\cdot \\ & \exp\left(-{\left(\frac{\lambda -\lambda_{B_{k}}-\Delta \lambda_{B_{k}}-\left(\delta \lambda \cdot p-\delta \lambda /2-N/2\right)}{\Delta \lambda_{{}_{FWHM}}}\right)}^{2}\right) \end{array}$$

where the *k*th sensor is described not only in terms of the number of sub-bands *N* and the codewords $a_{k}$ and $f_{k}$, specifying each sub-band binary amplitude (0,1) and phase value (0,π), but also some practical parameters are included: $\bar{R}$ is the reflectivity of each sensor; δλ is the spectral spacing between the slots; each sub-band linewidth $\Delta \lambda_{{}_{FWHM}}$ is determined by its the full width half maximum (FWHM) value; and $\Delta \lambda_{B_{k}}$ represents the induced wavelength shift. The sub-band shape was chosen as Gaussian for simplicity but, any other realizable shape is compatible with the encoding proposal.

In order to obtain a set of sensors mutually orthogonal with each other we take some concepts from optical orthogonal codes (OOC), which are binary codewords used in optical code division multiple access (OCDMA) communication systems; in our previous work, OOCs were used to shape the amplitude pattern of a SSFBG device. Here, we use the same concept with the addition of phase encoding to the reflecting sub-bands. This is represented in Equation (1), by the inclusion (or not) of the phase term exp(jπ) to the active sub-bands. An important practical requirement is that at least two consecutive active sub-bands are necessary in order to establish the phase difference between them in the interrogation stage. The effect achieved by adding phase encoding to the sensors can be seen as a new dimension in amplitude encoding, going from binary (0,1) to ternary encoding since a sign is added (0,+1,−1).

**Figure 2:**
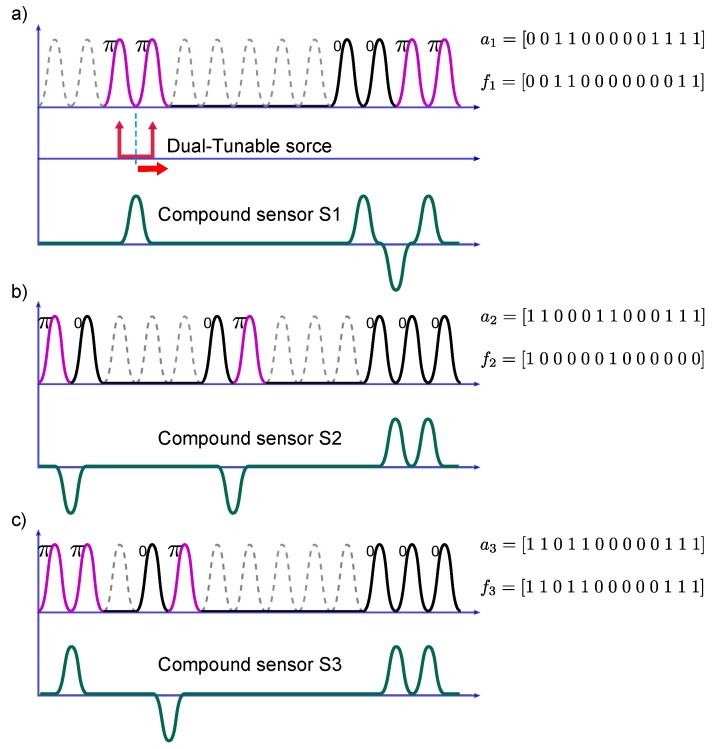
Set of three independent sensors designed according to the amplitude and phase codewords reported in Table 1. (**a**–**c**) depict the wavelength sub-band distribution for each super-structured fiber Bragg grating (SSFBG) device as well as their compound signals after interrogation through the dual-wavelength scanning.

A set of three amplitude and phase codewords is selected as exemplified in Table 1; these codewords are interpreted in increasing wavelength as described in Equation (1) and have a bit length of 13 positions, meaning that each SSFBG device features 13 slots to allocate the respective reflection sub-bands according to the codewords $a_{k}\;\mathrm{and}\;f_{k}$. However, due to the dual-wavelength interrogation principle, the retrieved information from each sensor consists of 12 sub-bands. For example, the first sensor (S1) is constructed by the codewords $a_{1}=\left[0\;0\;1\;1\;0\;0\;0\;0\;0\;1\;1\;1\;1\right]$ in amplitude, and $f_{1}=\left[0\;0\;1\;1\;0\;0\;0\;0\;0\;0\;0\;1\;1\right]$ in phase. Hence, it is composed of six sub-bands corresponding to the 1’s in the amplitude codeword $a_{1}$. In turn, the phase value for each sub-band is determined by the codeword $f_{1}$, that enables the factor exp(jπ) at the nonzero bit positions. Figure 2a–c depicts the equivalent sub-band distribution for the set of sensors in Table 1, as well as their compound interrogated signal. A change of phase between consecutive sub-bands is translated to a negative sign in the detected shape, likewise, two adjacent sub-bands without a phase change produce a detected signal with positive sign.

### 2.2. Interrogation

The interrogation method was described briefly in the Section 2.1. Now, we will detail this process from the theoretical perspective. To do so, it is necessary to define the source used to illuminate the sensors, and formulate its interaction with the sensing array. The dual-wavelength source is given by Equation (2).

(2) $$\begin{array}{cc} E_{in}\left(t\right) & =A_{a}\exp\left(i\left[\omega_{a}t+\varphi \left(t\right)\right]\right)+A_{b}\exp\left(i\left[\omega_{b}t+\varphi \left(t\right)\right]\right)\\ & =E_{a}\left(t\right)+E_{b}\left(t\right) \end{array}$$

where $\omega_{a}$ and $\omega_{b}$ are the general expressions for the two optical angular frequencies, and $A_{a},\;A_{b}$ represent each tone amplitude. These parameters depend on the modulation scheme used: with right-SSB modulation at a microwave frequency Ω for example, we get $\omega_{a}=\omega_{0}$, $\omega_{b}=\omega_{0}+\Omega$ and $A_{a}>A_{b}$. As a result, the interrogating signal $E_{in}$ is composed of two tones ($E_{a},\;E_{b}$ in Equation (2)) separated in frequency by $\Delta \omega =\omega_{a}-\omega_{b}$. $E_{in}$ is swept over the sensors operational range and reflected by the sensing elements in the network. The term φ(t) in Equation (2) describes the random variations in the optical phase of the CW source; this parameter is considered the same in the two tones because they are obtained through SSB modulation of the original source. Here, we are going to consider the full overlapping scenario between two neighboring sensors. This scheme is adequate to analyze the behavior of the network and can be extended to larger sensing networks. So, using a photo-detector to collect the reflected light from the sensing network, the gathered signal ($E_{s}$) would be written as in Equation (3):

(3) $$\begin{array}{cc} E_{s} & \propto \left[H_{a1}\exp\left(i\phi_{a1}\right)\cdot E_{a}\left(t\right)+H_{b1}\exp\left(i\phi_{b1}\right)\cdot E_{b}\left(t\right)\right]\\ & +\left[H_{a2}\exp\left(i\phi_{a2}\right)\cdot E_{a}\left(t-\Delta t\right)+H_{b2}\exp\left(i\phi_{b2}\right)\cdot E_{b}\left(t-\Delta t\right)\right] \end{array}$$

where $H_{ak}\exp\left(i\phi_{ak}\right)$ is the amplitude and phase response of the *k*th sensor in the lower sub-band $\omega_{a}$. In the same way, the term $H_{bk}\exp\left(i\phi_{bk}\right)$ corresponds to the upper sub-band $\omega_{b}$. Notice that the second sensor in the network gets delayed by Δt regarding sensor 1, due to the difference between their fiber paths.

In a ‘square-law’ detector, the detected photocurrent is proportional to:

(4) $$i\left(t\right)\propto E_{s}\cdot E_{s}^{*}$$

So, from Equations (3) and (4) the expected beating terms in the photodetector are listed in Equations (5)–(7).

First, we have the beating terms falling in the baseband region; these are represented in Equation set (5)

(5a) $$\begin{array}{cc} \left(H_{a1}^{2}+H_{a2}^{2}\right) & |E_{a}{|}^{2}+\left(H_{b1}^{2}+H_{b2}^{2}\right){\left|E_{b}\right|}^{2} \end{array}$$

(5b) Ha1Ha2exp(i(ϕa1−ϕa2))Ea(t)Ea*(t−Δt)x+exp(−i(ϕa1−ϕa2))Ea*(t)Ea(t−Δt)

(5c) Hb1Hb2exp(i(ϕb1−ϕb2))Eb(t)Eb*(t−Δt)x+exp(−i(ϕb1−ϕb2))Eb*(t)Eb(t−Δt)

Then, we will call *direct terms* to those terms created by the two interrogating bands being reflected from a unique sensor, they are expressed in Equation set (6).

(6a) Ha1Hb1exp(i(ϕa1−ϕb1))Ea(t)Eb*(t)x+exp(−i(ϕa1−ϕb1))Ea*(t)Eb(t)

(6b) Ha2Hb2exp(i(ϕa2−ϕb2))Ea(t−Δt)Eb*(t−Δt)x+exp(−i(ϕa2−ϕb2))Ea*(t−Δt)Eb(t−Δt)

On the other hand, the terms proceeding from two different sensors beating the two wavelengths are called the *cross terms*; they are represented in Equation set (7).

(7a) Ha1Hb2exp(i(ϕa1−ϕb2))Ea(t)Eb*(t−Δt)x+exp(−i(ϕa1−ϕb2))Ea*(t)Eb(t−Δt)

(7b) Hb1Ha2exp(i(ϕa2−ϕb1))Eb(t)Ea*(t−Δt)x+exp(−i(ϕa2−ϕb1))Eb*(t)Ea(t−Δt)

In order to develop the *direct* and *cross* terms, we need to compute the different products ($E_{a}\left(t\right)\cdot E_{b}\left(t\right)$) from Equations (6) and (7). It is important to note that the *direct* and *cross* terms oscillate at the fixed microwave frequency value Δω=Ω. The frequency response of the sensing network is then measured as the electro-optical frequency response at the modulation frequency (Ω). Hence, the detected photocurrent related to *direct* and *cross* terms is obtained as expressed in Equation set (8).

(8a) $$\begin{array}{cc} i_{d1} & =2A_{a}A_{b}H_{a1}H_{b1}\cos\left(\Delta \omega t+\left(\phi_{a1}-\phi_{b1}\right)\right) \end{array}$$

(8b) $$\begin{array}{cc} i_{d2} & =2A_{a}A_{b}H_{a2}H_{b2}\cos\left(\Delta \omega \left(t-\Delta t\right)+\left(\phi_{a2}-\phi_{b2}\right)\right) \end{array}$$

(8c) $$\begin{array}{cc} i_{x1} & =2A_{a}A_{b}H_{a1}H_{b2}\cos\left(\Delta \omega t+\left(\phi_{a1}-\phi_{b2}\right)+\varphi \left(\Delta t\right)+\omega_{b}\Delta t\right) \end{array}$$

(8d) $$\begin{array}{cc} i_{x2} & =2A_{a}A_{b}H_{b1}H_{a2}\cos\left(-\Delta \omega t+\left(\phi_{a2}-\phi_{b1}\right)+\varphi \left(\Delta t\right)+\omega_{a}\Delta t\right) \end{array}$$

Terms in Equation (8) represent the interaction between the dual-wavelength source and the sensing network for two generic wavelengths $\omega_{a}$ and $\omega_{b}$. We can conclude that direct terms, $i_{d}$ in Equation (8a,b), are present at the frequency Δω=Ω, and are affected by the phase of a unique sensing device. On the other hand, while cross terms, $i_{x}$, are present at the same frequency and affected by the phase component of the different sensors ($\phi_{ak}-\phi_{bk}$). They have two additional terms causing a phase delay: (φ(Δt)) is the phase noise inherent to the laser source, and ($\omega_{a,b}\Delta t$) describes the interferometric phenomenon caused by the cavity formed between two overlapping sub-bands. The effect of these terms on the sensors measurement is addressed in the next section.

**Figure 3:**
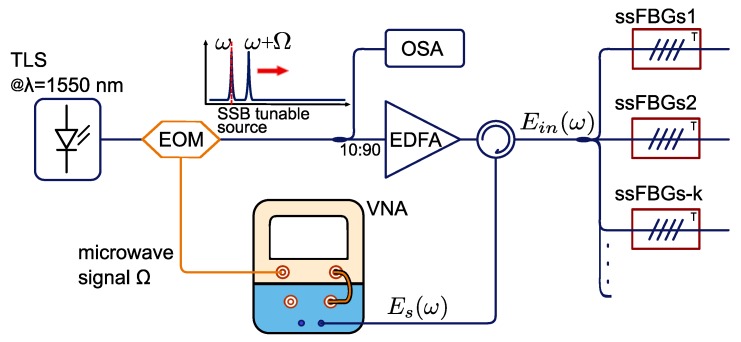
Interrogation setup to measure the frequency response of the sensing network. A dual-wavelength scanning source $E_{in}$ is created by the single side band modulation of a tunable laser at 1550 nm. The reflected waves from the sensing array $E_{s}$ are measured at the vectorial network analyzer (VNA) to compute the sensing network frequency response at the fixed microwave frequency Ω. OSA: optical spectrum analyzer.

The interrogation method to retrieve the phase and amplitude response from the sensors (see Figure 3) is performed by means of a vectorial network analyzer (VNA), which is used to measure the sensing network scattering parameter $S_{21}$ at a fixed microwave frequency (Ω). In this way, the complete electro-optical frequency response from the system H(Ω,λ) is obtained at the modulation frequency. This is known as a ‘zero-span’ mode measurement, meaning that the power of the sensing network frequency response is displayed versus time, at the fixed frequency. When we set this frequency to be Ω, the network frequency response is determined by the *direct* and *cross* terms, which are determined by the phase relationship between adjacent sub-bands of the K sensors present in the network ($\phi_{a_{k}},\;\phi_{b_{k}}$).

## 3. Experimental Results and Discussion

### 3.1. Wavelength Detection

**Figure 4:**
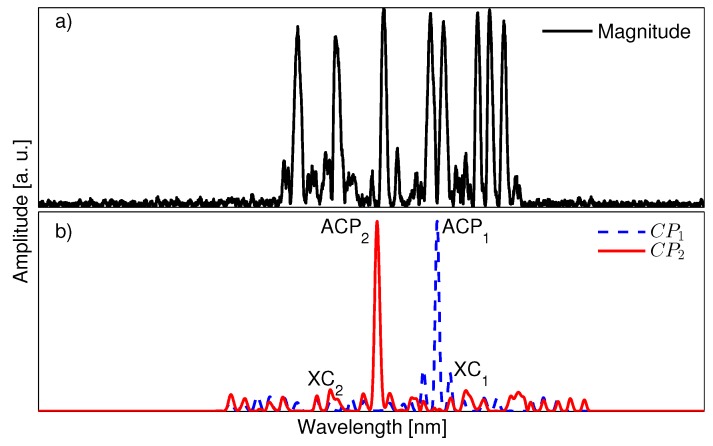
(**a**) Total reflected frequency response of the sensing network composed of two encoded fiber Bragg grating (FBG) sensors; and (**b**) the correlation product $CP_{k}$ computed for each sensor. The corresponding auto-correlation peak (ACP) value points to the matching wavelength location of each sensor in the spectrum. Cross correlation values (XC) are obtained for the remaining wavelength points.

After obtaining the frequency response from the sensing array, the next step is to assess the central wavelength that corresponds to each sensor in the network ($\lambda_{B_{k}}$). We use the correlation product between the sensing network frequency response H(Ω,λ), and the individual expected frequency response of each *k*th sensor in the network $H_{S_{k}}\left(\lambda^{\prime }\right)$. This can be written as in Equation (9).

(9) $$CP_{{}_{k}}\left(\lambda^{\prime }\right)=\int H\left(\Omega ,\lambda \right)\cdot H_{S_{k}}^{{}^{*}}\left(\lambda^{\prime }\right)\;\delta \lambda$$

where the correlation product $CP_{{}_{k}}$ is computed for each sensor over the wavelength operational range $\lambda^{\prime }$. The CP maximum point, known as the auto-correlation peak (ACP), occurs at the wavelength where the individual sensor and the compounded measurement coincide. Therefore, it indicates the central Bragg wavelength $\lambda_{B_{k}}$ for each sensor (see Figure 4 for illustration). The residual CP lower values constitute the cross-correlation signal (XC), which is constrained to a low value. This means that the sensor expected response does not match with the measured spectrum at those specific wavelength values. In Figure 4a, the magnitude component of the retrieved frequency response for a set of two sensors is shown. Figure 4b shows the corresponding ACP and XC values for the two sensors encoded in the compounded measurement.

**Figure 5:**
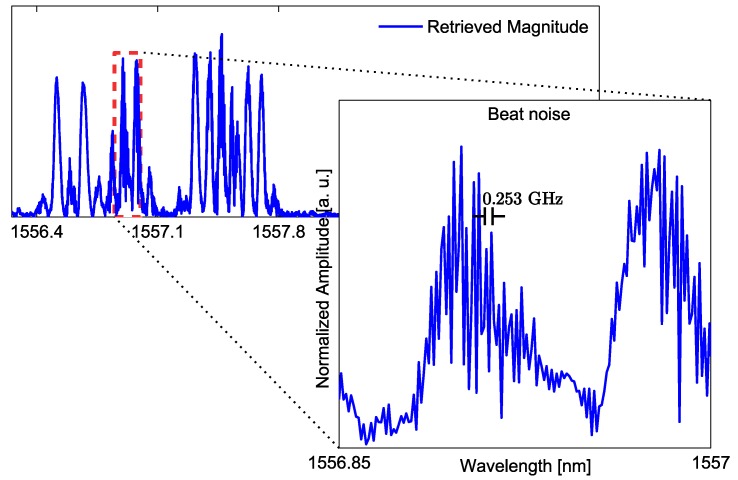
Zoomed-in region from a VNA readout. Ripple frequency due to overlapping between two sub-bands is measured to have a frequency of about 0.253 GHz, which is consistent with the placement distance between sensors of approximately 40 cm. (The x-axis represents the scanned wavelength and y-axis represents the normalized amplitude.)

Hence, the correlation product is the ideal mechanism to find the wavelength location of each sensor in the network. Still, it also offers an important advantage because this ‘moving product’ between each sensor’s expected shape and the compounded measurement actually bypasses unwanted measured components coming from the *cross* terms ($i_{x}$ in Equation (8c,d)). Cross terms, as described in Section 2.2, are produced when two sensors overlap and create a temporary cavity between the two reflection sub-bands. The travel time of the cavity is expressed in Equation (8c,d) by the terms $\omega_{a}\Delta t\;\mathrm{and}\;\omega_{b}\Delta t$. The effect of the cavity can be observed in the measurement as a fluctuating signal with a ripple frequency that depends on the travel time distance between the two sensors. For instance, Figure 5 shows a readout from an overlapping scenario between three encoded SSFBG sensors; the difference between ‘clean’ sub-bands (without ripple effect) and the overlapped sub-bands is noticeable. The subplot details the ripple effect by zooming into two affected reflection sub-bands. The ripple frequency is determined by measuring the peak to peak fluctuation in the plot, obtaining a frequency of 0.253 GHz equivalent to a round-trip travel time of 3.952 ns (i.e., the two overlapping sensors conform a cavity of approximately 40 cm).

### 3.2. SSFBG Device Manufacturing

**Figure 6:**
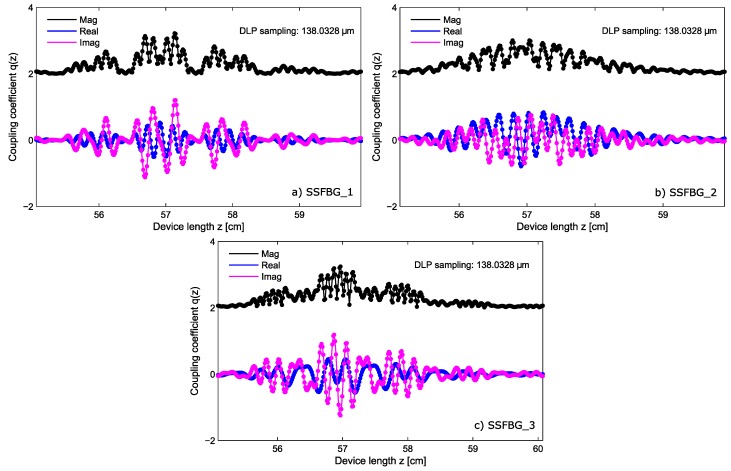
Complex coupling coefficient q(z), for the three designed SSFBG sensing devices. The magnitude as well as the real and imaginary components for each sensing device are shown in (**a**–**c**). The amplitude of the coupling coefficient is proportional to the ac index profile of the grating.

**Figure 7:**
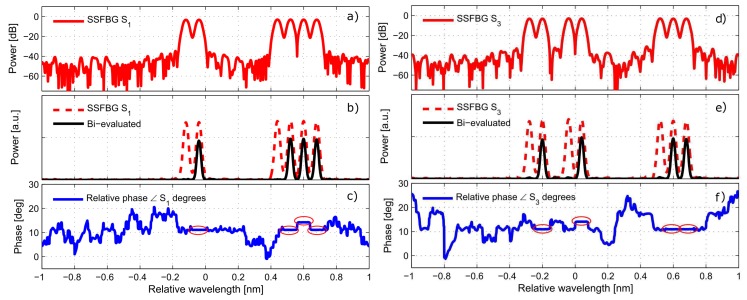
Design for the super-structured sensor $S_{1}$ and $S_{3}$. (**a**,**d**) Represents the magnitude spectrum in dB for the first and third designed sensor; (**b**,**e**) the linear representation of the magnitude spectrum and their bi-evaluated shape; and (**c**,**f**) the relative phase of the devices $S_{1}$, $S_{3}$, i.e., the phase relationship between consecutive sub-bands of the device.

The manufacturing of the super-structured devices as designed in Section 2.1 (see the codewords in Table 1) was performed by using the discrete layer peeling (DLP) synthesis method. The spectral profiles used as targets are those ones depicted in Figure 2, The design was performed with a linewidth of FWHM=40 pm, spacing between sub-bands of δω=10 GHz, and reflectivity set to the 50% in order to use a parallel configuration setup. As a result, from the synthesis method we obtain the complex index profile q(z), shown for each sensor in Figure 6, where the coupling coefficient is depicted in its magnitude as well as real and imaginary components for each sensor. For each of the devices the index profile is sampled at ∼138 μm. The analysis of the complex index profile is presented in Figure 7 for the devices $S_{1}$ and $S_{3}$. This figure shows the sensor amplitude (in dB and linear representation), their bi-evaluated amplitude (which is the result of dual-wavelength interrogation of the sensors), and their phase component, displaying the relative phase change required between consecutive sub-bands.

The manufacturing process is performed point by point through ultra-violet (UV) laser beam exposure using the phase mask (PM) method to produce the compounded Bragg pattern. Hence, the optical fiber was exposed twice for each sampling point, to a UV beam focused to a diameter of ∼80 μm, with a power of ∼48 mW. In this way the construction of the grating is controlled by changing the relative alignment between consecutive exposures, maintaining the average UV flux and therefore the effective refractive index [9]. The manufacturing system available in the Photonics Research Labs (PRL) is able to construct the fast changes of each custom shape in the q(z) profile. Each sampled point in Figure 6 represents the theoretical location of a double UV exposure. Furthermore, each q(z) profile was truncated at 5% of its amplitude, obtaining a total length for the sensing devices of $S_{1}=4.858$ cm, $S_{2}=4.775$ cm, and $S_{3}=4.983$ cm. Values of q(z) below this threshold are not significant in the device manufacturing. The physical length of the SSFBG devices presented here is considerably longer than for conventional FBG sensors. Therefore, they are suited for applications with uniform temperature along the size of the sensors. The temperature behavior of the different sub-bands in super-structured sensors is described in [17].

It is important to note, in Figure 7b, that the ‘bi-evaluated’ response for each sensor is the expected result after the interrogation of the SSFBG devices with the dual-wavelength source as explained in Figure 2. In the same way, the phase relationship is retrieved between consecutive sensor sub-bands (meaning that the measured phase component is not the actual phase of the component but the relative phase between the two interrogated points). Therefore, the correlation product should be performed between the ‘bi-evaluated’ measured response and the ‘bi-evaluated’ expected response.

**Figure 8:**
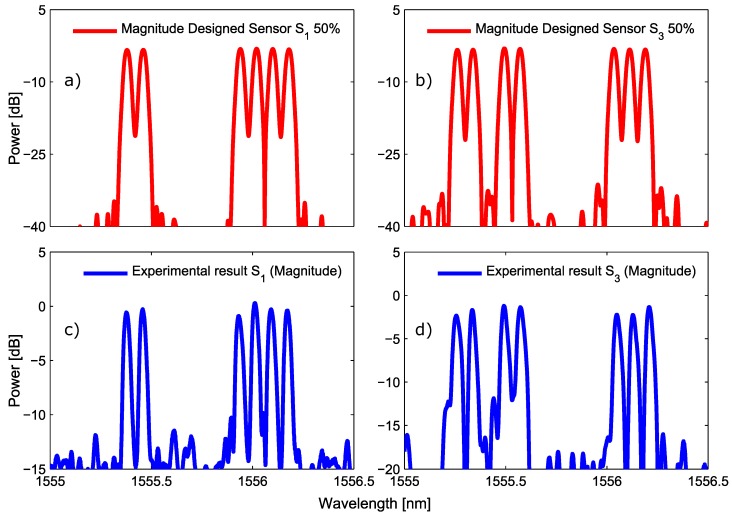
The measured magnitude of the manufactured SSFBG is depicted in (**c**,**d**) for the devices $S_{1}$ and $S_{3}$. The designed magnitude of this sensors is shown in (**a**,**b**) for comparison.

Figure 8 shows, in magnitude, the comparison between the designed and the experimental measurement for the SSFBG devices $S_{1}$ and $S_{3}$. In the measurement, performed with an optical vector analyzer (OVA), the reflectivity value slightly surpasses the 3dB value due to the manual adjustment of the manufacturing setup. However, the manufactured devices show a very good agreement regarding the designed ones.

### 3.3. Experimental Validation

After successful manufacturing of the sensing devices. The experiment with three overlapping sensors was carried out and the setup is the one proposed in Figure 3. A narrow-linewidth tunable laser source (*Yenista TUNICS T100R*) was swept over the operating range of the sensors at 1 nm/s. During the sweeping, the laser was SSB modulated at 10 GHz to get the dual-wavelength interrogation signal.

The dual-wavelength source triggers the VNA to measure in time the complete network frequency response. Additionally, an optical spectrum analyzer (OSA) is used to monitor the interrogating signal.

The sensors are stabilized in temperature and strain shifted in order to create the overlapping scenario. At each point of the experiment, the readout from the sensing network is captured at the VNA and stored in the computer to get each sensor central wavelength.

**Figure 9:**
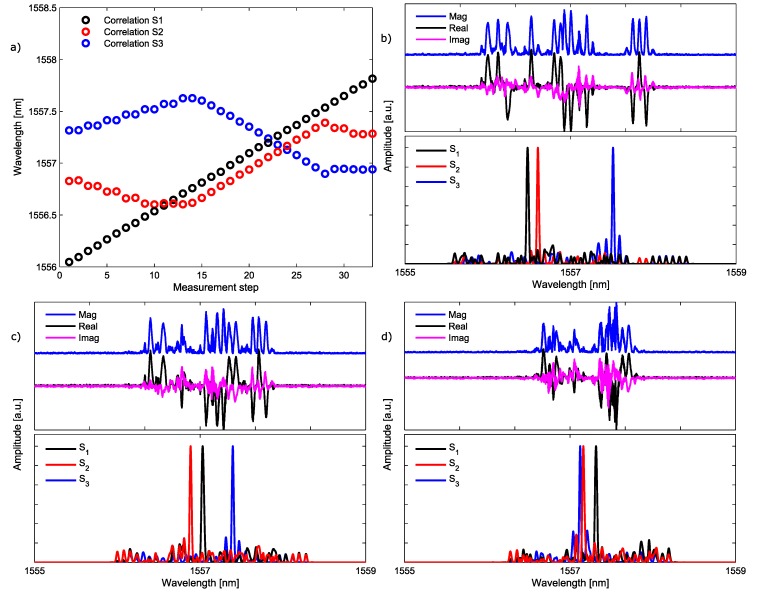
Each sensor’s central wavelength is depicted for the whole overlapping scenario between the three sensors in (**a**). (**b**–**d**) Three intermediate captures of the system; the upper plot depicts the sensing network readout (magnitude, real and imaginary components) while the lower plot presents the retrieved central wavelength obtained for each sensor after running the correlation algorithm. Magnitude representations have an offset in order to facilitate the visualization.

As mentioned in Section 3.1, the demodulation method, employed to retrieve the central wavelength of each sensing element in the network, consists of the correlation product between the bi-evaluated network frequency response measured in the VNA at 10 GHz, and the expected frequency response for each manufactured sensor. Figure 9b–d depict three different readouts from the system in their upper plots, and the corresponding correlation product obtained for each one of the three sensors in the lower ones. Figure 9a depicts the central wavelength, measured for the three sensors at every step of the complete overlapping scenario. This plot shows that our measurement approach is able to totally identify the spectral position of three sensors that overlap in the same spectral region. Consequently, we can extrapolate the behavior of the proposed code-division multiplexing scheme to an hybrid CDM–WDM system, in which, for example, three sensors can be placed at each WDM channel. Thus, an enhancement in the number of sensors, proportional to the number of codewords used, 3 in our case, can be achieved.

The last stage of the experimental validation consists in the assessment of the error induced to the system when we add a number of sensors that actually share the same spectral range. In order to characterize the amount of induced error, we need to remove the effect of the additional sensors interfering within the readout. Consequently, we performed an experiment that recreates the overlapping scenario, so that we could measure both the individual contribution from each sensor and the compounded readout when two or three sensors are interacting with each other. In this way, we also neglect the human error from the measurements since we are taking the individual and compounded contributions at the same instant along the same experiment.

**Figure 10:**
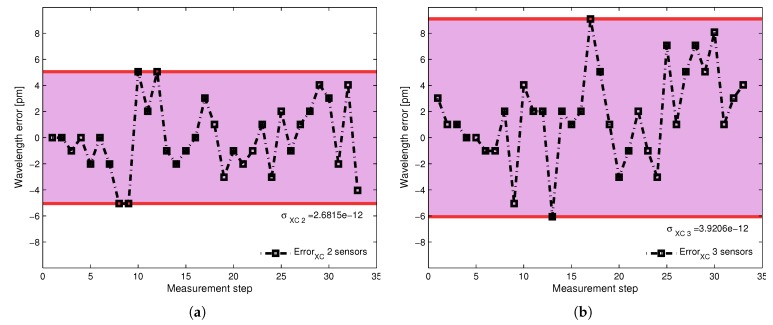
Error found for the isolated sensor after comparison with the detected wavelength in presence of a second sensor (**a**), and after comparison with two additional sensors interacting in the same spectral range (**b**).

Hence, the individual wavelength was measured for the sensing device $S_{3}$; this measured wavelength was then contrasted against its measured wavelength in presence of sensor $S_{1}$ and finally against its wavelength when sensors $S_{1}$ and $S_{2}$ where sharing the spectral region. The point by point difference between the individual measured wavelength and the wavelength obtained in presence of an additional sensor is depicted in Figure 10a. Figure 10b shows the point-by-point difference when two additional sensors are interacting with the individual sensor. The standard deviation due to the cross-correlation between two sensors is $\sigma_{xc2}=2.68$ pm, and due to the interaction between three sensors, $\sigma_{xc3}=392$ pm.

## 4. Conclusions

In this paper we have proposed an encoding technique to add a multiplexing dimension to the traditional WDM approach in FBG sensing networks and enhance the number of sensors that can be deployed. The encoding approach proposed here is intended to add information to each sensor by modifying its amplitude and phase profiles. Therefore, we matched the design of the sensor’s amplitude and phase profiles with the interrogation technique in such a way that they complemented each other. The interrogation technique uses a dual wavelength source and a vectorial network analyzer to retrieve the differential profile from each sensing device in the network. This technique and its interaction with the sensors was theoretically developed in order to better understand the measured terms from the network frequency response.

Taking advantage of the possibilities of manufacturing specialized super-structured FBG devices, we fabricated the proposed encoded sensors with very specific profiles in amplitude and phase. This allowed us to perform an experimental validation of the proposed technique, showing that the system is capable of identifying each sensor wavelength even when three sensors are merging into each other spectrally. We also addressed the error added to the network due to the existence of overlapping between two or three devices. This additional error has a very low impact in the measurement. The worst case of standard deviation was 3.9 pm, when three sensors were sharing the same spectral region. We showed that the off-line detection procedure averages the ripple frequency caused by overlapping sensor sub-bands. Future work will involve the development of additional off-line procedures to decrease the cross-talk noise gain due to the interference between sensors.

## Figures and Tables

**Table 1:** Set of three sensors represented by their amplitude and phase codewords.

Sensor	Codeword	
S1	$a_{1}$	[0011000001111]
	$f_{1}$	[0011000000011]
S2	$a_{2}$	[1100011000111]
	$f_{2}$	[1000001000000]
S3	$a_{3}$	[1101100000111]
	$f_{3}$	[1101100000111]
